# Distinction of Different Colony Types by a Smart-Data-Driven Tool

**DOI:** 10.3390/bioengineering10010026

**Published:** 2022-12-24

**Authors:** Pedro Miguel Rodrigues, Pedro Ribeiro, Freni Kekhasharú Tavaria

**Affiliations:** CBQF—Centro de Biotecnologia e Química Fina–Laboratório Associado, Escola Superior de Biotecnologia, Universidade Católica Portuguesa, Rua de Diogo Botelho 1327, 4169-005 Porto, Portugal

**Keywords:** petri-plates, colonies, machine-learning models, discrimination

## Abstract

Background: Colony morphology (size, color, edge, elevation, and texture), as observed on culture media, can be used to visually discriminate different microorganisms. Methods: This work introduces a hybrid method that combines standard pre-trained CNN keras models and classical machine-learning models for supporting colonies discrimination, developed in Petri-plates. In order to test and validate the system, images of three bacterial species (*Escherichia coli*, *Pseudomonas aeruginosa*, and *Staphylococcus aureus*) cultured in Petri plates were used. Results: The system demonstrated the following Accuracy discrimination rates between pairs of study groups: 92% for *Pseudomonas aeruginosa* vs. *Staphylococcus aureus*, 91% for *Escherichia coli* vs. *Staphylococcus aureus* and 84% *Escherichia coli* vs. *Pseudomonas aeruginosa*. Conclusions: These results show that combining deep-learning models with classical machine-learning models can help to discriminate bacteria colonies with good accuracy ratios.

## 1. Introduction

Evaluation of the number of viable microorganisms in a sample is a commonly used method in most microbiology laboratories. The method consists of counting visible colonies on agar plates and calculating the number of colony-forming units per mL (or gram) of the sample. For example, it is widely used for food, clinical, environmental, and drug safety testing. The counting of bacteria is usually carried out manually, and is, therefore, subjective and error-prone [1]. At present, automatic digital counters are common in laboratories and some have highly efficient automatic counting methods, which have replaced manual counting methods.

Although the counting of visible colonies on agar plates is the most commonly used method to assess bacterial populations, with the advantage of only considering the counts of viable cells [2], it is time-consuming, laborious and requires at least 24 h or more for visible colonies to form. This can be a considerable limitation in some situations, such as quality control of certain foods and in clinical settings, where fast results are required so that actions can rapidly be implemented.

One important factor in cell counting is the analyst’s ability to see colonies distinctly. Colony morphology is used to select bacteria as phenotypically different. This is normally carried out by visual inspection, and the selected parameters are often colony size, color, texture, edge, and elevation, according to the colony morphology protocol emitted by the American Society for Microbiology [3].

In a previous work, a software capable of semi-automatically quantifying the number of colonies in Petri plates from a digital image was developed [4]. This method did not, however, automatically distinguish different colony types. Thus, in the present work, we attempted to include this distinguishing characteristic. Therefore, three bacterial species (*Escherichia coli*, *Pseudomonas aeruginosa*, and *Staphylococcus aureus*) that represent the predominant pathogenic microorganisms in a variety of settings—food [5], clinical [6] and environmental [7]—were used to evaluate and develop our solution/software to support colony discrimination. Table 1 shows the the current state-of-art on colony-distinguishing methods based on machine-learning (ML) models.

## 2. Methodology

**Figure 1:**
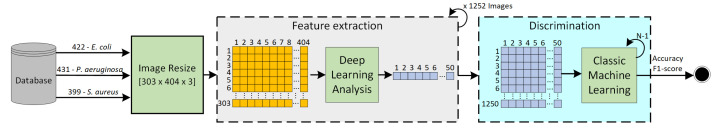
Methodology workflow.

In this section, all the procedures are described. The microbiological analysis and the image database are presented and, after that, the deep and classical machine-learning analysis of images is explained. Figure 1 presents a summary of the whole methodology procedure.

### 2.1. Microbiological Analysis and Image Database

Plates containing *Escherichia coli*, *Pseudomonas aeruginosa* and *Staphylococcus aureus* isolates from our center’s internal collection were cultivated aerobically at 37 °C, for 24 h, in Trypto-Casein Soy Agar™ (TSA, BIOKAR Diagnostics, Allonne, France) using the spread-plate technique (0.1 mL of the diluted samples). All experiments were carried out in triplicate. Colony enumeration was performed and the number of colonies was recorded and posteriorly attributed to each image of the database.

The final dataset [12] consists of about 1252 labeled Petri images with 422 colonies of *Escherichia coli*, 431 of *Pseudomonas aeruginosa* and 399 of *Staphylococcus aureus*. The color images were acquired by a smartphone camera with 12 megapixels [3024 × 4032 × 3]. For more details, consult the previous authors’ published paper [4].

### 2.2. The Deep and Classical Machine-Learning Analysis

To verify the suitability of the Image dataset for building deep-learning models that can obtain a total of 50 features from each colony for image-based microorganism recognition, we evaluated the performance of the following standard, pre-trained 31 CNN keras models [13]: Xception; VGG16; VGG19; ResNet50; ResNet50V2; ResNet101; ResNet101V2; ResNet152; ResNet152V2; InceptionV3; InceptionResNetV2; MobileNet; MobileNetV2; DenseNet121; DenseNet169; DenseNet201; EfficientNetB0; EfficientNetB1; EfficientNetB2; EfficientNetB3; EfficientNetB4; EfficientNetB5; EfficientNetB6; EfficientNetB7; EfficientNetV2B0; EfficientNetV2B1; EfficientNetV2B2; EfficientNetV2B3; EfficientNetV2S; EfficientNetV2M; EfficientNetV2L. For more details please check the Keras default models at https://keras.io/api/applications/, accessed on 20 November 2022.

Due to the relatively high resolution of all images, the samples were scaled down to [303 × 404 × 3] to reduce the computation time and guarantee proper aspect ratios. Thus, the patches of each neural network architecture were resized to match the default input layer size. The output layer of each used standard CNN keras models [13], and was also replaced by a dense layer with 50 units and softmax as the activation function to obtain, as output, in a blinding feature extraction process, 50 features from each colony to serve as vector inputs for several classical ML models: decision trees (DT), support-vector machines (SVM), K-nearest neighbors (KNN), multi-layer perceptron (MLP) and three ensemble classifiers (please check Table 2 for more details). The models’ performance was evaluated within a leave-one-out-cross-validation procedure, a well-known process that allows for the use of all datasets for testing, without leakage between train and test sets.

In this work, the feature extraction and the classification were carried out in a cloud-based service, the Google Colaboratory. The software code was developed in Python-Jupyter Notebook for machine-learning and deep-learning operations within a virtual machine with two Intel Xeon CPUs both at 2.20 GHz, 100 GB of storing, and 13 GB of Ram.

The evaluation metric for colony detection was based on the Accuracy and *F*1-*score* [14]. Accuracy shows how many cases were correctly labelled out of all the cases, and is defined as,

(1) $$Accuracy=\frac{TruePositives+TrueNegatives}{TruePositives+TrueNegatives+FalsePositives+FalseNegatives}\times 100\%$$

where a TruePositive is an outcome in which the MP model correctly predicts a positive class, a TrueNegative is an outcome where the model correctly predicts the negative class, a FalsePositive is an outcome where the model incorrectly predicts the positive class and, finally, FalseNegative is an outcome where the model incorrectly predicts the negative class [14].

The *F*1-*score* is the harmonic mean of precision and recall and can be defined as,

(2) $$F1-score=2\times \frac{precision\times recall}{precision+recall}\times 100\%$$

where precision and recall are, respectively,

(3) $$precision=\frac{TruePositives}{TruePositives+FalsePositives}$$

and

(4) $$recall=\frac{TruePositives}{TruePositives+FalseNegatives}$$

Thus, if the *F*1-*score* is high, both the precision and recall of the classifier indicate good results [14].

## 3. Results and Discussion

**Figure 2:**
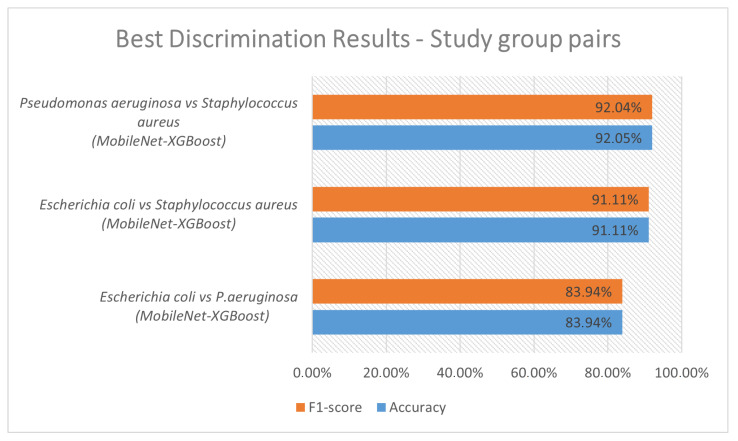
Best discrimination results between study group pairs.

By analyzing Table 3, some considerations regarding the classification results between pairs of study groups are revealed. Accuracies higher than 84% were obtained for all pairs, with at least one combination of deep and classical machine-learning methods. The combination of classifiers MobileNet-XGBoost provided the best results for all study pair classifications; in this way, it was shown to be a good candidate combination for differentiating colonies. The XGBoost was shown to be the most effective classical machine-learning classifier, as 81% (82 of 93) of the best combinations of deep and classical machine-learning have XGBoost as a classifier. The group pairs comparisons that involved *Staphylococcus aureus* achieved high Accuracy and *F*1-*score* rates, above 91%. One of the explanations for these results is that *Staphylococcus aureus* produces yellow colonies [15] on a plate, which are very typical and differentiated from the *Escherichia coli* and *Pseudomonas aeruginosa* that produce beige colonies on a plate [16,17]. As *Escherichia coli* and *Pseudomonas aeruginosa* colonies are both beige on a plate, the problem of differentiating each becomes more difficult for the classifiers. Even so, the proposed methods achieved good ratios of Accuracy and *F*1-*score* ≈ 84% on *Escherichia coli* vs. *Pseudomonas aeruginosa* discrimination. The graphic of Figure 2 shows the best discrimination results between the study groups. The results are in line with those found in the state-of-art literature (please check Table 1) and provides us with a good indication that, if we continue to improve and refine the algorithm, we can build an even more helpful, powerful, and robust tool for this purpose.

## 4. Conclusions

This work introduced a preliminary method that combines standard CNN keras models and classical machine-learning models to support colony discrimination, developed in Petri-plates. In order to test and validate the system, images of three bacterial species (*Escherichia coli*, *Pseudomonas aeruginosa*, and *Staphylococcus aureus*) cultured in Petri plates were presented to the CNN models’ entries to extract 50 image features to feed classical machine-learning models within a leave-one-out-cross validation procedure. The system demonstrated good accuracy discrimination rates between pairs of study groups: 92% for *Pseudomonas aeruginosa* vs. *Staphylococcus aureus*, 91% for *Escherichia coli* vs. *Staphylococcus aureus* and 84% *Escherichia coli* vs. *Pseudomonas aeruginosa*. The presented preliminary results showed that a combination of deep-learning models and classical machine-learning models can help to discriminate bacteria colonies in Petri-plates. Tools, such as the one developed in the present work, are really valuable in ascertaining different colony types in a single step, using a general, whole-purpose medium instead of several selective and/or differential media, rendering the process time-consuming, expensive, and prone to errors due to the increased manipulation steps required by the operator. Furthermore, differential colony counting is quite useful, since most analyzed samples in a microbiology setting are not pure-culture, but mixed cultures involving more than one bacterial species. In future work, the dataset should be extended to more bacteria colony types to evaluate the system’s ability to discriminate other species and should include a set of pictures containing a mixture of colonies to evaluate the accuracy of the method in a mixed/complex sample. Additionally, the deep and classical machine-learning models should be refined to improve the system’s performance.

## Figures and Tables

**Table 1:** State-of-the-art papers.

Ref.	Year	ML Model	Comparison Group	Accuracy
[8]	2021	SVM	*E. coli* vs. *S. aureus* vs. *S. Typhimurium* vs. *E. faecium* vs. *P. aeruginosa*	93.3%
[9]	2017	CNN	33 bacteria comparison (all the bacteria used in this study are included)	97.24%
[10]	2019	CNN	33 bacteria comparison (all the bacteria used in this study are included)	98.22%
[11]	2022	Linear Discriminant	*E. coli* vs. *E. coli-β* vs. *S. aureus* vs. *methicillin-resistant S. aureus* vs. *P. aeruginosa* vs. *E. faecalis* vs. *K. pneumoniae* vs. *C. albicans*	92%

**Table 2:** Used classical machine-learning classifiers and optimal parameters.

ML Model		Optimal Parameters
DT	Medium Tree	Maximum number of splits = 150 & criterion = “gini”
SVM	Radial Basis	Cost = 1 & gamma = 2
KNN	Balltree	Number of neighbors = 3
MLP	1 input layer	activation function = “relu”
		training algorithm = “adam”
	1 hidden layer	L2 regulation term = 1
		fullyConnectedLayer = 3
	1 output layer	hidden layer neurons = 100
Ensemble	Random Forest (RF)	Maximum number of splits = 100 & criterion = “gini”
	Bagged Trees (BagT)	Maximum number of splits = 150 & criterion = “gini”
	XGBoost	boosted trees to fit = 150
		learning rate = 0.1
		max depth of the tree = 6
		L2 regulation term = 1

**Table 3:** Summary of the best discrimination results between study group pairs.

*Escherichia coli* vs. *Pseudomonas aeruginosa*			*Escherichia coli* vs. *Staphylococcus aureus*			*Pseudomonas aeruginosa* vs. *Staphylococcus aureus*		
Classifiers	*Accuracy*	*F*1-*Score*	Classifiers	*Accuracy*	*F*1-*Score*	Classifiers	*Accuracy*	*F*1-*Score*
Xception-DT	76.79%	76.76%	Xception-XGBoost	81.49%	81.47%	Xception-XGBoost	81.33%	81.26%
VGG16-XGBoost	77.26%	77.26%	VGG16-XGBoost	84.90%	84.89%	VGG16-XGBoost	84.70%	84.67%
VGG19-XGBoost	71.86%	71.85%	VGG19-XGBoost	84.04%	84.04%	VGG19-XGBoost	84.94%	84.93%
ResNet50-XGBoost	77.96%	77.95%	ResNet50-XGBoost	86.97%	86.97%	ResNet50-XGBoost	86.75%	86.74%
ResNet50V2-XGBoost	76.20%	76.17%	ResNet50V2-XGBoost	82.10%	82.09%	ResNet50V2-XGBoost	87.11%	87.11%
ResNet101-BagT	76.91%	76.89%	ResNet101-XGBoost	85.51%	85.49%	ResNet101-XGBoost	88.43%	88.42%
ResNet101V2-XGBoost	74.79%	74.79%	ResNet101V2-XGBoost	75.52%	75.50%	ResNet101V2-XGBoost	78.43%	78.37%
ResNet152-XGBoost	79.02%	78.99%	ResNet152-XGBoost	86.11%	86.11%	ResNet152-XGBoost	86.99%	86.99%
ResNet152V2-XGBoost	75.15%	75.15%	ResNet152V2-XGBoost	78.20%	78.19%	ResNet152V2-XGBoost	80.96%	80.94%
InceptionV3-XGBoost	75.38%	75.37%	InceptionV3-XGBoost	76.49%	76.49%	InceptionV3-RF	77.83%	77.68%
InceptionResNetV2-XGBoost	74.91%	74.91%	InceptionResNetV2-XGBoost	74.30%	74.30%	InceptionResNetV2-XGBoost	79.28%	79.24%
**MobileNet-XGBoost**	**83.94%**	**83.94%**	**MobileNet-XGBoost**	**91.11%**	**91.11%**	**MobileNet-XGBoost**	**92.05%**	**92.04%**
MobileNetV2-XGBoost	78.90%	78.88%	MobileNetV2-XGBoost	85.75%	85.75%	MobileNetV2-XGBoost	88.55%	88.54%
DenseNet121-KNN	79.60%	79.60%	DenseNet121-KNN	83.80%	83.77%	DenseNet121-XGBoost	86.51%	86.49%
DenseNet169-XGBoost	79.48%	79.48%	DenseNet169-KNN	84.17%	84.15%	DenseNet169-KNN	84.82%	84.76%
DenseNet201-XGBoost	79.60%	79.58%	DenseNet201-XGBoost	84.41%	84.41%	DenseNet201-XGBoost	86.14%	86.15%
EfficientNetB0-XGBoost	68.35%	68.24%	EfficientNetB0-XGBoost	74.91%	74.91%	EfficientNetB0-XGBoost	80.84%	80.78%
EfficientNetB1-XGBoost	70.57%	70.54%	EfficientNetB1-XGBoost	78.93%	78.93%	EfficientNetB1-KNN	83.13%	83.06%
EfficientNetB2-XGBoost	72.22%	72.22%	EfficientNetB2-XGBoost	82.10%	82.09%	EfficientNetB2-XGBoost	81.57%	81.53%
EfficientNetB3-XGBoost	67.76%	67.75%	EfficientNetB3-XGBoost	69.79%	69.77%	EfficientNetB3-XGBoost	75.90%	75.82%
EfficientNetB4-XGBoost	76.55%	76.55%	EfficientNetB4-XGBoost	78.81%	78.80%	EfficientNetB4-XGBoost	83.73%	83.71%
EfficientNetB5-XGBoost	71.51%	71.51%	EfficientNetB5-XGBoost	80.63%	80.63%	EfficientNetB5-BagT	83.49%	83.45%
EfficientNetB6-XGBoost	66.47%	66.47%	EfficientNetB6-XGBoost	71.86%	71.87%	EfficientNetB6-XGBoost	76.39%	76.31%
EfficientNetB7-XGBoost	75.62%	75.61%	EfficientNetB7-XGBoost	84.77%	84.77%	EfficientNetB7-XGBoost	87.83%	87.82%
EfficientNetV2B0-XGBoost	75.38%	75.38%	EfficientNetV2B0-XGBoost	83.68%	83.67%	EfficientNetV2B0-XGBoost	86.63%	86.60%
EfficientNetV2B1-XGBoost	75.85%	75.83%	EfficientNetV2B1-XGBoost	84.04%	84.03%	EfficientNetV2B1-XGBoost	87.47%	87.45%
EfficientNetV2B2-XGBoost	75.85%	75.85%	EfficientNetV2B2-KNN	80.63%	80.52%	EfficientNetV2B2-XGBoost	84.10%	84.05%
EfficientNetV2B3-XGBoost	79.95%	79.95%	EfficientNetV2B3-XGBoost	83.68%	83.68%	EfficientNetV2B3-XGBoost	86.51%	86.50%
EfficientNetV2S-XGBoost	70.93%	70.92%	EfficientNetV2S-XGBoost	75.03%	75.01%	EfficientNetV2S-XGBoost	77.47%	77.41%
EfficientNetV2M-XGBoost	65.42%	65.42%	EfficientNetV2M-XGBoost	70.89%	70.89%	EfficientNetV2M-XGBoost	67.47%	67.45%
EfficientNetV2L-BagT	63.89%	63.86%	EfficientNetV2L-XGBoost	72.59%	72.59%	EfficientNetV2L-XGBoost	72.41%	72.31%

## Data Availability

The data presented in this study are openly available in FigShare at doi, reference number 10.6084/m9.figshare.20109377.v2.
